# Efficacy and Safety of One Anastomosis Gastric Bypass Versus Roux-en-Y Gastric Bypass for Obesity: a Meta-analysis and Systematic Review

**DOI:** 10.1007/s11695-022-06401-5

**Published:** 2022-12-23

**Authors:** Xianting Li, Xu Hu, Chendong Fu, Lang Han, Ming Xie, Shurui Ouyang

**Affiliations:** 1 Department of Digestive Disease Hospital, Affiliated Hospital of Zunyi Medical University, No. 149, Dalian Road, Huichuan District, Zunyi, 563000 Guizhou China; 2 Department of General Surgery, Affiliated Hospital of Zunyi Medical University, No. 149, Dalian Road, Huichuan District, Zunyi, 563000 Guizhou China

**Keywords:** One anastomosis gastric bypass, Roux-en-Y gastric bypass, Obesity, Meta-analysis

## Abstract

The objective of this review is to systematically review the efficacy and safety outcomes of one anastomosis gastric bypass (OAGB) with Roux-en-Y gastric bypass (RYGB). From inception to July 4, 2022, a systematic literature search was performed using PubMed, Embase, and Cochrane Library for randomized clinical trials comparing OAGB with RYGB in obesity. A meta-analysis performed using the RevMan 5.4.1 software evaluations was completed. We identified 1217 reports; after exclusions, eight trials with a total of 931 patients were eligible for analysis. Compared with RYGB, OAGB had multiple advantageous indexes. Examples include percent of excess weight loss (%EWL) at 12 months (*P* = 0.009), body mass index (BMI) at 2 years (*P* < 0.00001), early postoperative complication (*P* = 0.04), remission of dyslipidemia (*P* < 0.0001), and operative time (*P* < 0.00001). No significant statistical difference was observed in BMI at 6 months, %EWL at 6 months, BMI at 12 months, percent of excess body mass index loss (%EBMIL) at 2 years, BMI at 5 years, intraoperative complications, late postoperative complications, remission of type 2 diabetes mellitus, and dyslipidemia or gastroesophageal reflux disease remission between OAGB and RYGB. OAGB is no less effective than RYGB; no significant differences in weight loss efficacy were observed, and more large and long-term randomized controlled trials are needed to verify this. In addition, studies have shown that OAGB has a shorter operation time, fewer early postoperative complications, and a shorter learning curve, making it easier for young surgeons to perform.

## Introduction

According to the World Health Organization, as early as 2016, more than 1.9 billion adults were overweight and more than 650 million obese [1]. A study predicted that by 2025, the global male obesity rate will reach 18%, while the female obesity rate will exceed 21% [2]. Obesity may be accompanied by complications, such as hyperglycemia, hypertension, and dyslipidemia, all of which endanger personal health [3, 4]. Currently, the number of obese people in the world is increasing, and the health problems associated with it have attracted more attention. How to effectively lose weight has become a critical health concern. The methods of weight loss include diet adjustment, exercise, drugs, and surgical treatment. Surgical treatment is currently considered the most effective and durable weight loss method [5–7].

Currently, bariatric surgery includes Roux-en-Y gastric bypass (RYGB), one anastomosis gastric bypass (OAGB), laparoscopic sleeve gastrectomy (LSG), and sleeve gastrectomy with jejunojejunostomy/enteral bypass (SG-JJB). There are many types of bariatric surgery, and the subjective factors of doctors have a great influence on the choice of surgical methods. According to the 2018 IFSO (International Federation for The Surgery of Obesity and Metabolic Disorders) survey of bariatric surgeries, LSG, RYGB, and OAGB are the three most common surgeries [8]. LSG is mainly suitable for people with a low overweight BMI because the original access to the gastrointestinal tract is maintained as much as possible and there are fewer complications. However, since the small intestine is not bypassed, its weight loss effect is usually not satisfactory for super obese people. Both RYGB and OAGB procedures involve gastrointestinal reconstruction and bypassing a part of the jejunum. For patients with a higher BMI, most scholars consider these types of surgeries [9, 10]. Between 2003 and 2013, RYGB has been the most commonly used bariatric surgery worldwide [11]. OAGB has been officially reported since 2001 and has grown rapidly in recent years [12]. RYGB is a type of weight loss surgery that involves creating a small gastric pouch from the proximal end of the stomach. The small gastric pouch is then connected with the distal jejunum [9]. In OAGB, a tubular gastric pouch is first established, and the distal end of the gastric pouch is connected to the jejunum at a distance of 150–250 cm from the ligament of Treitz [9]. Both surgical approaches reduce the volume of the stomach and bypass the jejunum to limit food intake and absorption. RYGB involves creating a small gastric pouch but bypasses the jejunum less, while OAGB involves creating a slightly longer gastric pouch; however, it bypasses the jejunum more. However, the two methods of gastrointestinal reconstruction are different; hence, the weight loss effect of the two surgical methods may differ as may the incidence of related complications. There have been previous comparative studies on the two surgical methods, and it is believed that the postoperative effect of OAGB is comparably worse to that of RYGB, and that it has the advantages of shorter operation time and fewer complications; however, most of them are retrospective or small sample size studies [13–16].

We will further select randomized controlled trials and include new studies to conduct a meta-analysis and systematic review of OAGB and RYGB to compare the weight loss effect, metabolic syndrome improvement, and incidence of related complications in obese patients after different bariatric procedures, among other differences.

## Material and Methods

Referencing according to the Meta Writing Guidelines (PRISMA) published in 2009 [17].

### Inclusion Criteria

The inclusion criteria are as follows: (1) randomized controlled trials comparing OAGB and RYGB; (2) include previous English-language articles; (3) the follow-up time must be ≥ 1 month; (4) follow-up content must include one of the following: BMI, percent of excess weight loss (%EWL), or percent of excess BMI loss (%EBMIL).

### Exclusion Criteria

The exclusion criteria are as follows: (1) articles were withdrawn; (2) articles on animal experiments; (3) full text not.

### Search Strategy

We searched the PubMed, Embase, and Cochrane Library databases, under the title and abstract contexts using the keywords “one anastomosis gastric bypass,” “mini gastric bypass,” “single anastomosis gastric bypass,” or “omega loop gastric bypass,” and “Roux-en-Y gastric bypass” in pairwise combinations. The search period spanned from the start of the study until July 4, 2022. Two members independently conducted the search process and included articles. A third reviewer was consulted if there was a disagreement concerning the inclusion of an article.

### Data Extraction

Data was extracted independently by two members and included the following:

1. Basic characteristics: author, year, country, sample size, age, preoperative BMI, operation time, hospital stay, follow-up time, and endpoints
2. Surgery characteristics: gastric pouch volume, biliopancreatic limb length, food limb length, and intraoperative complications
3. Postoperative characteristics: BMI, %EWL, %EBMIL, remission of comorbidities, and postoperative complications (serious complications and general complications) [18]

If verification is required by a third reviewer, we may choose to contact the author or editor for further information.

### Risk of Bias

Two authors independently performed the quality assessment. The quality of randomized controlled trials was evaluated with the Cochrane collaboration’s tool to assess the risk of selection including random sequence generation, allocation concealment, blinding of participants and personnel, blinding of outcome assessment, incomplete outcome data, selective reporting, and other biases. The risk of bias was assessed for these seven items. The decision of whether it was “low risk of bias,” “high risk of bias,” or “unclear” was made according to the risk of bias assessment criteria [19].

### Data Analysis

Endpoint definitions in the literature included BMI [weight/height^2^] and %EWL = [(initial weight) (postoperative follow-up weight)]/[preoperative overweight weight] × 100%. The target weight was set to be the weight corresponding to a BMI equal to 25 kg/m^2^. %EBMIL = [ΔBMI/(preoperative initial BMI 25)] × 100%.

Statistical analysis was performed using RevMan 5.4.1. The risk ratio (RR) was used as an effect analysis statistic for categorical variables, and the mean difference (MD) was used as an effect analysis statistic for numerical variables. All statistics were calculated using a 95% confidence interval (CI). When *P* > 0.05 and *I*^2^ < 50%, the fixed effects model was used, whereas when *P* ≤ 0.05 or *I*^2^ ≥ 50%, the random effects model was used.

### Study Selection

**Fig. 1:**
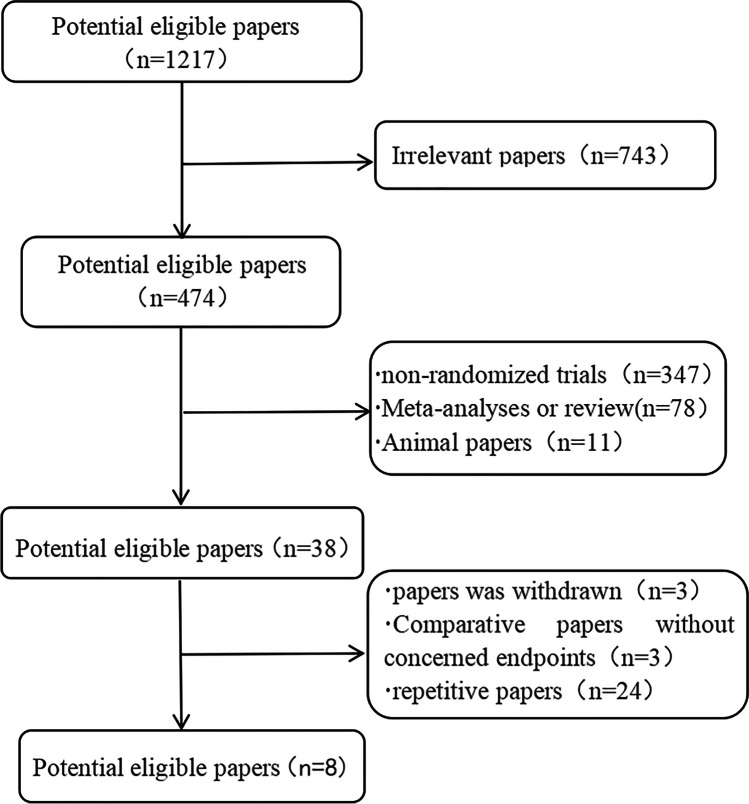
Flow chart for searching articles

A total of 1217 articles were initially retrieved through PubMed, Cochrane, and Embase databases. We excluded 743 articles comparing non-OAGB and RYGB procedures, leaving 474. After screening the inclusion and exclusion criteria, 38 articles were retained. Three of them were the same articles withdrawn from the three databases, one article was the same article that did not include follow-up data in the three databases, and there were 24 duplicate articles. Finally, eight randomized controlled trials were included in this study (Fig. 1).

### Study Characteristics and Risk of Bias

**Fig. 2:**
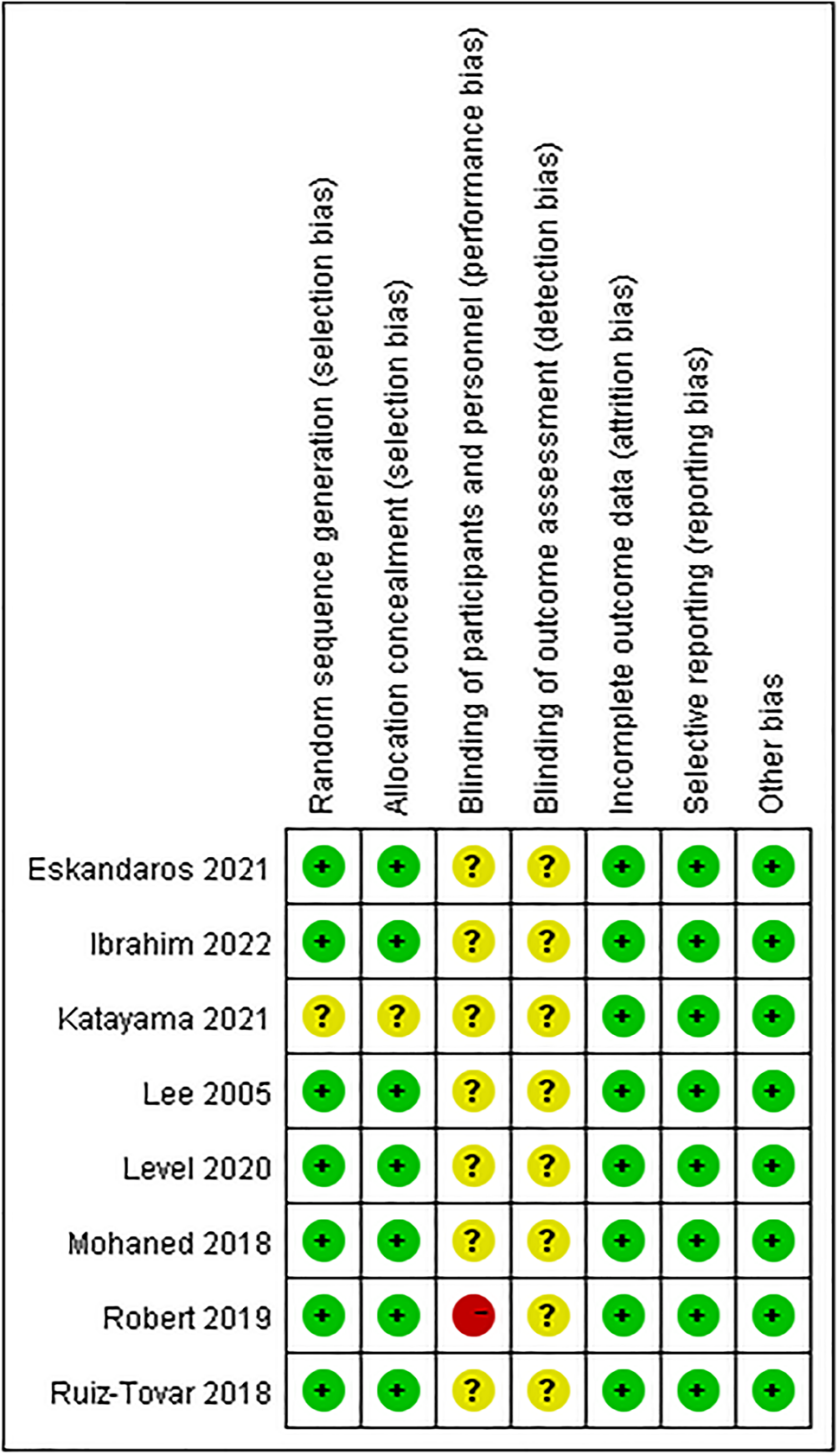
Risk of bias assessment of included studies. Note: “ + ” low risk of bias; “?” unclear; “-” high risk of bias

A total of eight prospective randomized controlled trials were included in our meta-analysis [20–27]. The papers were published between 2005 and 2022, and a total of 931 patients participated. The basic characteristics of the included studies are summarized in Table 1, with mean ages ranging from 30.7 to 45 years and mean preoperative BMI ranging from 42.6 to 53.5 kg/m^2^. In the study providing information on metabolic syndrome, there were 288 patients with type 2 diabetes, 321 patients with hypertension, 188 patients with hyperlipidemia, 101 patients with gastroesophageal reflux syndrome, and 133 patients with apnea syndrome. The surgical technique characteristics of the included studies are summarized in Table 2. Figure 2 shows the risk of bias in the included studies. Almost all the articles had risk of bias due to blinding, which was not described in the text.

**Table 1:** Basic characteristics of included studies (OAGB/RYGB)

Author, year	Robert, 2019	Mohaned, 2018	Lee, 2005	Ruiz-Tovar, 2018	Level, 2020	Eskandaros, 2021	Katayama, 2021	Ibrahim, 2022
Country	France	Egypt	China	Spain	Venezuela	Egypt	Brazil	Egypt
Sample	117/117	30/30	40/40	180/184	9/19	40/40	10/10	30/35
Female (%)	85/91	90 (F total)	67.5/70	75/75	100/100	52.5/50	100/90	86.67/60
Mean age (year)	44.4 ± 11.4/42.6 ± 10.2	31.3 ± 8.0/32.7 ± 7.3	30.7 ± 8.4/31.1 ± 9.1	43.8 ± 11.5/45 ± 11.3	37.5 ± 6.6/36.8 ± 9.3	36 ± 10/36 ± 11	43.2 ± 3.7/43.1 ± 3.9	37.7 ± 9.9/36.9 ± 10.2
Preoperative BMI (kg/m^2^)	43.8 ± 6.1/43.9 ± 5.1	45.5 ± 5.3/44.1 ± 4.7	44.8 ± 8.8/43.8 ± 4.8	45 ± 4.1/45.3 ± 3.2	42.9 ± 5.5/42.6 ± 5.9	49.78 ± 3.40/50.01 ± 3.50	43.2 ± 3.7/43.1 ± 3.9	53.5 ± 9.4/52.3 ± 5.1
Operative time (min)	85 ± 35/111 ± 42	95 ± 10/137 ± 10	147.7 ± 46.7/205.0 ± 60.5	-	113.3 ± 41.2/143.7 ± 21.85	133.80 ± 17.95/172.78 ± 11.02	78.0 ± 13.4/125.5 ± 10.9	119.7 ± 17.7/143.1 ± 16.9
Hospital stay (day)	4–5/4–6	-	5.5/6.9	-	3/3	-	-	-
Follow-up time	2 years	6 months, 1 year	1, 2 years	1, 2, 5 years	1, 6 months, 1, 5 years	6月, 1 year	1, 3 months	3, 6, 12 months
Endpoint	Overweight weight loss	GERD score	Weight loss	Overweight weight loss	Weight loss	PAGI-SYM score	Weight loss	Weight loss

“-” means not reported in the papers

**Table 2:** Surgical technical characteristics

Surgery		Robert, 2019	Mohaned, 2018	Lee, 2005		Ruiz-Tovar, 2018	Level, 2020	Eskandaros, 2021		Katayama, 2021	Ibrahim, 2022
	Gastric pouch volume (mL)	-	25–35	-		-	-	-	-	-	
OAGB	Biliopancreatic limb length (cm)	200	200	200		250–350	200	BMI < 45 45 < BMI < 50 BMI > 50	180 200 220	200	200
RYGB	Gastric pouch volume (mL)	30	-	15–20		-	20	-		-	-
	Food limb length (cm)	150	150	BMI < 49 BMI > 50	100 150	150	150	BMI < 45 45 < BMI < 50 BMI > 50	120 135 150	100	150
	Biliopancreatic limb length (cm)	50	50	50		100	100	BMI < 45 45 < BMI < 50 BMI > 50	60 80 100	50	60

“-” means not reported in the papers

## Results

### Postoperative Weight Loss

**Fig. 3:**
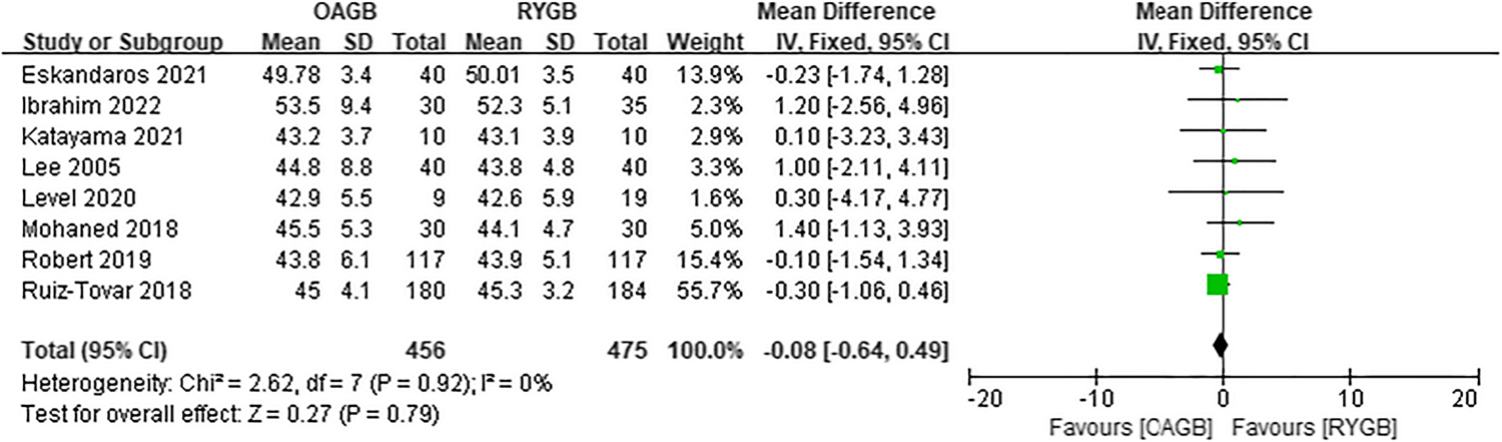
Preoperative BMI

**Fig. 4:**
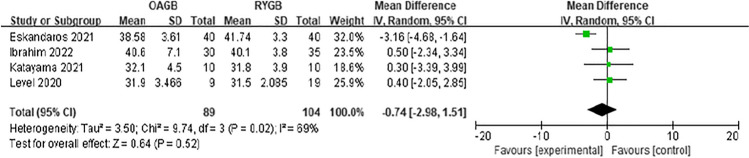
BMI at 6 months after surgery

**Fig. 5:**
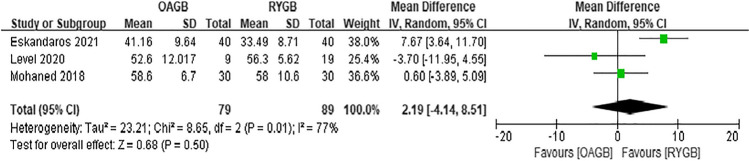
%EWL at 6 months after surgery

**Fig. 6:**
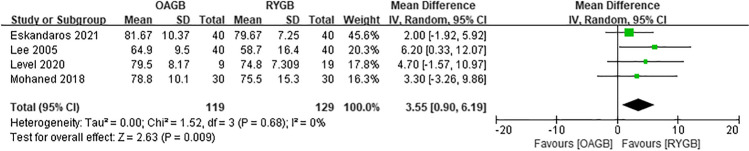
%EWL at 12 months after surgery

**Fig. 7:**
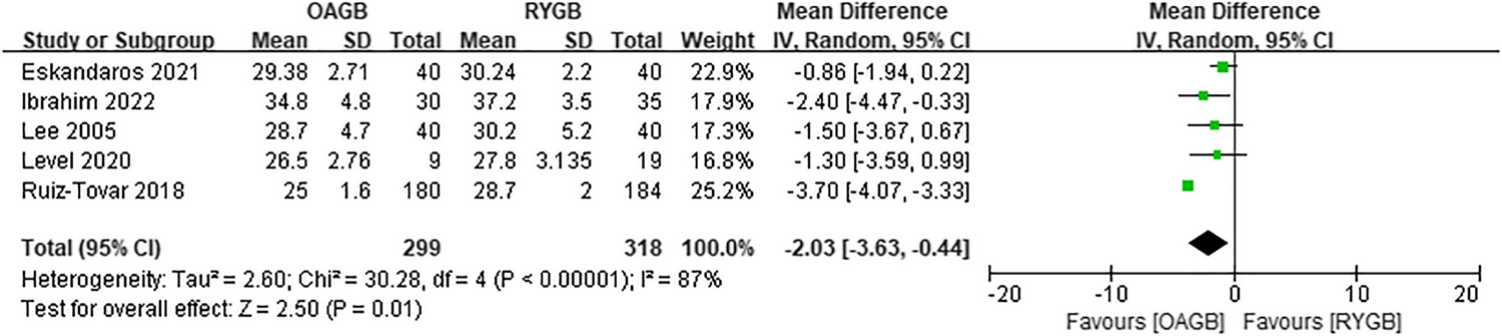
BMI at 12 months after surgery

**Fig. 8:**
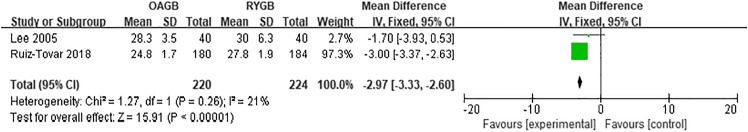
BMI at 2 years after surgery

**Fig. 9:**
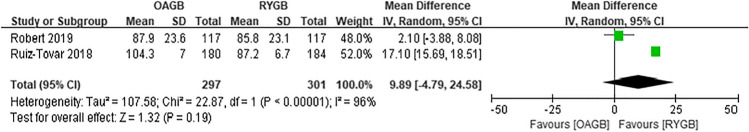
%EBMIL at 2 years after surgery

**Fig. 10:**
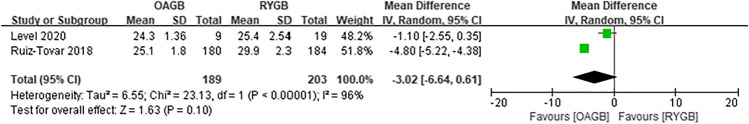
BMI at 5 years after surgery

Since the outcome measures described in each article were inconsistent, we decided to perform a meta-analysis of postoperative weight loss outcomes using BMI, %EWL, or EBMIL. First, the preoperative BMI was analyzed. In the eight included articles, there was low heterogeneity in preoperative BMI between OAGB and RYGB, and the difference was not statistically significant (MD =  − 0.08, *I*^2^ = 0%, 95% CI =  − 0.64 to 0.49, *P* = 0.79) (Fig. 3). Regarding the BMI at 6 months after surgery, four articles were included, and the difference was not statistically significant (MD =  − 0.74, *I*^2^ = 69%, 95% CI =  − 2.98 to 1.51, *P* = 0.52) (Fig. 4). Regarding the %EWL at 6 months after surgery, four articles were included, and the difference was not statistically significant (MD = 2.19, *I*^2^ = 77%, 95% CI =  − 4.14 to 8.51, *P* = 0.50) (Fig. 5). A total of four articles were included for %EWL at 12 months after surgery, and OAGB was considered superior to RYGB (MD = 3.55, *I*^2^ = 0%, 95% CI = 0.90 to 6.19, *P* = 0.009) (Fig. 6). A total of five articles were included for BMI at 12 months after surgery, and it was considered that OAGB had better weight loss effect than RYGB (MD =  − 2.03, *I*^2^ = 87%, 95% CI =  − 3.63 to − 0.44, *P* = 0.01) (Fig. 7). Two articles were included for BMI at 2 years after surgery, and the weight loss effect of OAGB was better than that of RYGB (MD =  − 2.97, *I*^2^ = 21%, 95% CI =  − 3.33 to − 2.60, *P* < 0.00001) (Fig. 8). Two articles were included for the %EBMIL comparison between OAGB and RYGB at 2 years after surgery and there was no statistical significance (MD = 9.89, *I*^2^ = 96%, 95% CI =  − 4.79 to 24.58, *P* = 0.19) (Fig. 9). Two articles were included for BMI at 5 years after surgery and there was no statistical significance (MD =  − 3.02, *I*^2^ = 96%, 95% CI =  − 6.64 to 0.61, *P* = 0.10) (Fig. 10).

### Complications and Remission of Comorbidities

**Fig. 11:**
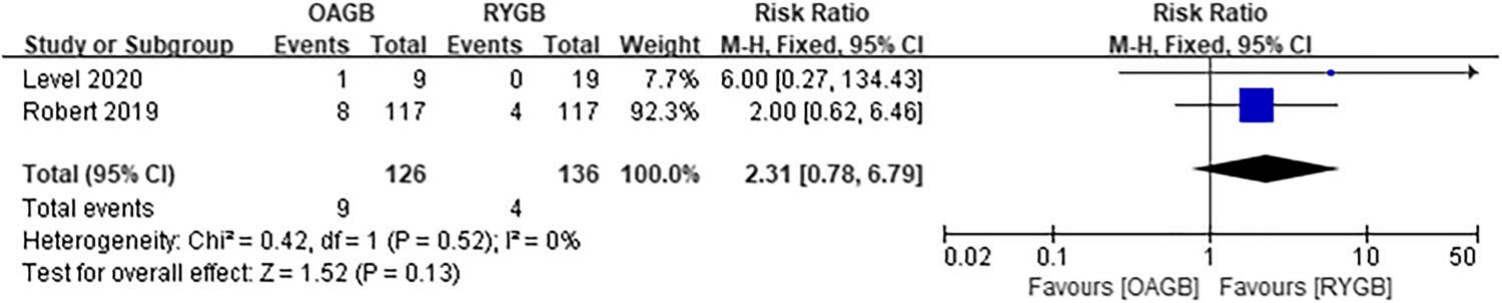
Intraoperative complications

**Fig. 12:**
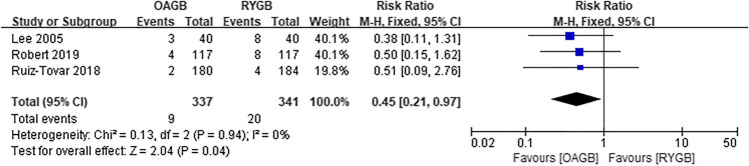
Early postoperative complications

**Fig. 13:**
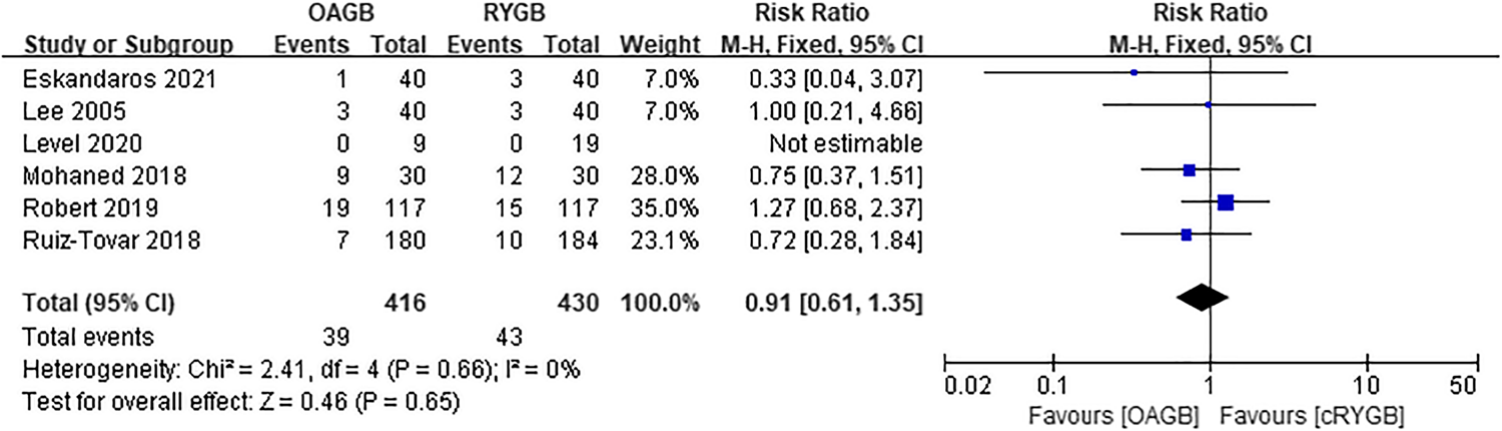
Late postoperative complications

A total of two articles were included for intraoperative complications, and there was no statistical significance between OAGB and RYGB (RR = 2.31, *I*^2^ = 0%, 95% CI = 0.78 to 6.79, *P* = 0.13) (Fig. 11). Regarding intraoperative complications, the OAGB group included four cases of bleeding, three cases of intestinal injury, and two cases of iatrogenic suture of nasogastric tube. The RYGB group included three cases of bleeding and one case of intestinal injury. Three articles were included in the early postoperative complications and there was statistical significance. It was observed that OAGB had fewer complications (RR = 0.45, *I*^2^ = 0%, 95% CI = 0.21 to 0.97, *P* = 0.04) (Fig. 12). Among the early postoperative complications, the OAGB group included three serious complications (one of which required surgery) and six cases of general complications. The RYGB group included eight serious complications (two of which required surgery) and twelve cases of general complications. Late complications included all complications that were not staged in the article. Regarding late complications from the two surgical methods, a total of six articles were included and there was no difference (RR = 0.91, *I*^2^ = 0%, 95% CI = 0.61 to 1.35, *P* = 0.65) (Fig. 13). Among those with late postoperative complications, the OAGB group included three serious complications (two of which required surgery), 17 general complications, and 19 unspecified stage complications. The RYGB group included 13 serious complications (nine of which required surgery), 15 general complications, and 15 unspecified stage complications. In addition, according to Robert et al., there were 24 cases of RYGB and 42 cases of OAGB surgery-related adverse events up to 2 years of follow-up.

**Fig. 14:**
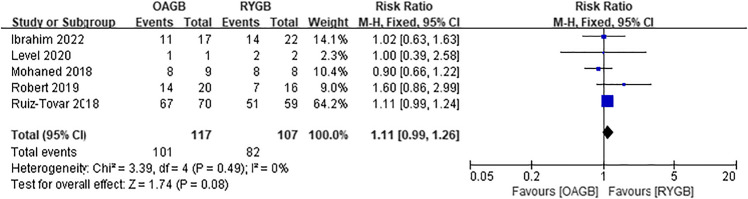
Postoperative diabetes remission

**Fig. 15:**
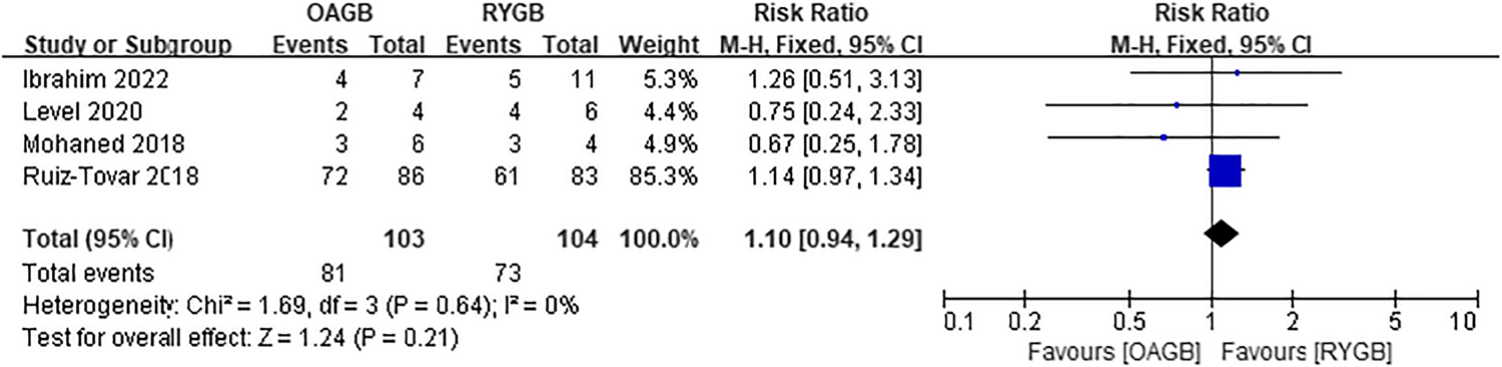
Postoperative hypertension remission

**Fig. 16:**
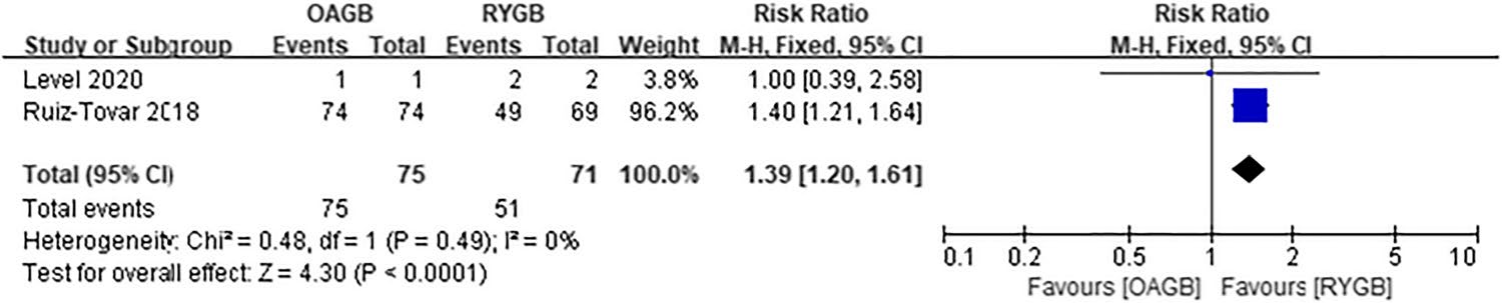
Postoperative hyperlipidemia remission

**Fig. 17:**
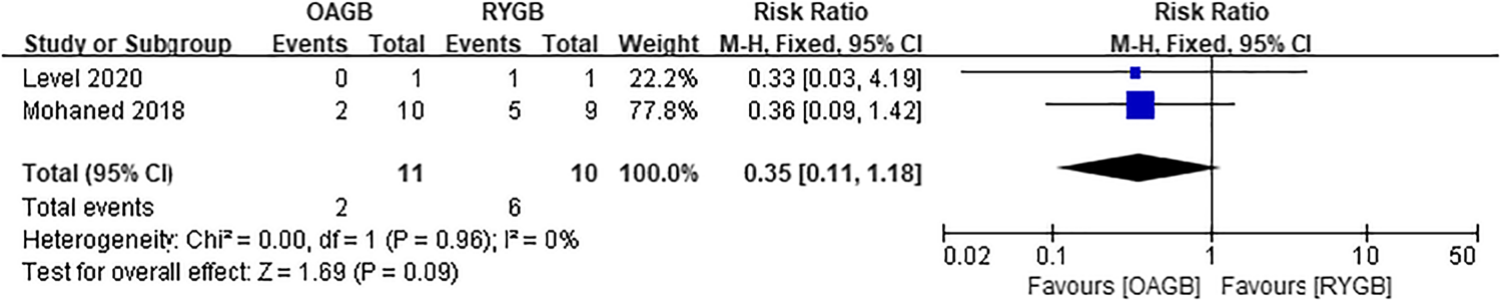
Postoperative GERD remission

Diabetes, hypertension, hyperlipidemia, and gastroesophageal reflux disease (GERD) were all obesity-related complications in our meta-analysis, and postoperative remission of all complications was based on the study’s last follow-up time. Postoperative diabetes remission included patients with partial and complete remission, which included five articles, and there was no difference between the two surgical methods (RR = 1.11, *I*^2^ = 0%, 95% CI = 0.99 to 1.26, *P* = 0.08) (Fig. 14). Four articles were included for postoperative hypertension remission, and there was no difference between the two surgical methods (RR = 1.10, *I*^2^ = 0%, 95% CI = 0.94 to 1.29, *P* = 0.21) (Fig. 15). Two articles were included for postoperative hyperlipidemia remission with statistical significance, and OAGB had a higher remission rate (RR = 1.39, *I*^2^ = 0%, 95% CI = 1.20 to 1.61, *P* < 0.0001) (Fig. 16). Two articles were included for postoperative GERD remission, and no difference between the two surgical methods was observed (RR = 0.35, *I*^2^ = 0%, 95% CI = 0.11 to 1.18, *P* = 0.09) (Fig. 17).

### Operation Time, Revision, and Mortality

**Fig. 18:**
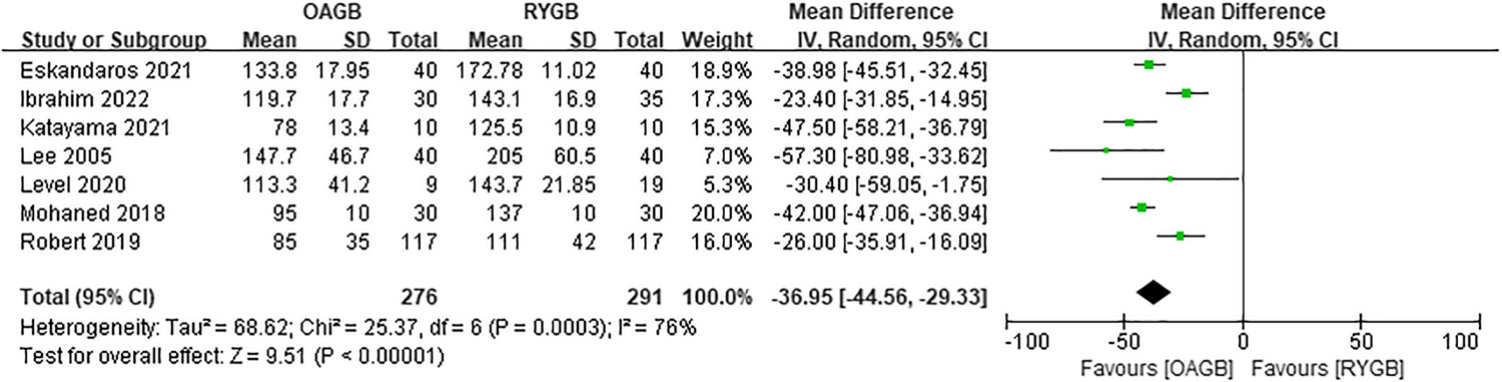
Operation time

A total of seven articles were included for operation time, and there was statistical significance. The operation time of OAGB was significantly shorter than that of RYGB (MD =  − 36.95, *I*^2^ = 76%, 95% CI =  − 44.56 to − 29.33, *P* < 0.00001) (Fig. 18).

Regarding postoperative revision surgery, Robert et al. excluded four patients from the population who changed from OAGB to RYGB [20]. In the study of Ruiz-Tovar et al., two patients changed to RYGB due to bile acid reflux after OAGB, and three patients underwent revision surgery because of weight gain after RYGB [23]. Two patients died in the study by Robert et al.; hence, they were excluded from the study [20]. There were no deaths in the remaining studies.

## Discussion

RYGB was first described in 1967, and an IFSO survey showed that RYGB was the most common bariatric procedure from 2003 to 2013 [11, 28]. According to the 2018 IFSO survey results, OAGB was considered the third most popular bariatric surgery [8]. Previous studies have suggested that the OAGB learning curve is shorter than that of RYGB, and that the learning curve is closely related to surgical complications [29–31]. However, there is no consistent confirmation of the effect and comorbid remission rate of the two bariatric surgeries. Many articles have shown that the weight loss effect of OAGB is not as worse as that of RYGB; however, the sample size of the comparison articles is small, the randomized trials are few, and they mainly focused on the comparison of weight loss effect, with less research on postoperative complications. Therefore, we included randomized controlled trials of OAGB or RYGB bariatric surgery in obese patients for systematic review and meta-analysis to evaluate the efficacy and safety of different bariatric surgeries. We hope to provide a reference for bariatric surgeons when making surgical decisions.

### Postoperative Weight Loss

We analyzed preoperative BMI, and found no significant difference among the included studies, implying that comparing postoperative BMI is feasible for evaluating the effect of weight loss. We selected multiple indicators for comparison at each time point since the indicators reflecting the effect of weight loss were different in each study. In the meta-analysis of %EWL at 6 months, BMI at 6 months and 12 months, %EBMIL at 2 years, and BMI at 5 years, there was no significant difference in weight loss between OAGB and RYGB. The results of the %EWL and 2-year BMI analysis at 12 months after surgery showed that the weight loss effect of OAGB was more satisfactory than that of RYGB. In the included study, Ruiz-Tovar et al. believed that OAGB had a better weight loss effect since the biliopancreatic limbs were longer than required during digestive tract reconstruction [23]. According to the 2018 World Bariatric and Metabolic Surgery consensus, obese patients undergoing OAGB should have a biliopancreatic limb length of 150 cm when their BMI is < 50 and a biliopancreatic limb length of 200 cm when their BMI is > 50. The biliopancreatic limb length of obese patients undergoing RYGB should be between 50 and 150 cm, while the biliopancreatic limb length of Ruiz-Tovar et al.’s study participants for OAGB was 250–350 cm [9]. The duodenum and jejunum are important organs for food digestion and absorption; however, the biliopancreatic limb is a bypass segment of the small intestine, and ingested food does not pass through this channel, limiting nutrient absorption. In the long biliopancreatic limb, the absorption of more nutrients is restricted [32].

Some previous systematic reviews and meta-analyses have also shown that the weight loss effect of OAGB is not inferior to that of RYGB, and it is believed that this may be due to the longer indwelling biliopancreatic limbs during reconstruction. However, the included articles are few and bias is not well controlled; hence, conclusions are questionable [33, 34]. Similarly, we should realize that the length of the biliopancreatic limbs using OAGB is not as long as possible. While considering the effect of weight loss, the length of the common channel should be ensured to reduce the incidence of postoperative malnutrition [35, 36]. RYGB surgery has shorter biliopancreatic limbs than OAGB but a smaller stomach volume and more obvious restriction on food intake. Therefore, the long-term weight loss effects of the two surgery methods may be similar. This is also consistent with our study, which observed no significant differences between the two surgical methods in terms of %EBMIL at 2 years and BMI at 5 years. Therefore, the standard of biliopancreatic limb length and the influence of gastric pouch size on patients can be further explored and standardized in large-scale clinical studies to achieve the effect of weight loss without causing malnutrition.

### Postoperative Complications

Studies on early postoperative complications suggest that OAGB has fewer complications than RYGB, with lower a heterogeneity, larger sample size, and higher reliability. The study found that RYGB had more major complications than OAGB, among which anastomotic leakage was the most common. OAGB had no anastomotic leakage, while RYGB had a total of five. There are four possible reasons for the high incidence of anastomotic leakage in RYGB. First, OAGB has only one gastro-jejunal anastomosis, while RYGB has gastro-jejunal anastomosis and jejunal-jejunal anastomosis, increasing the incidence of anastomotic leakage in RYGB [9]. Second, whereas both RYGB and OAGB are involved in removing the right gastroepiploic and left gastroepiploic arteries, only one or two branches of the left gastroepiploic artery remained when the gastric pouch was formed after RYGB [37, 38]. This leads to differences in the blood supply of the anastomotic stoma between the two surgical methods, and since gastrojejunostomy leakage is more common in bariatric surgery, anastomotic leakage caused by blood supply cannot be excluded [39]. Third, RYGB is more difficult to operate than OAGB. Moreover, the learning curve is longer and negatively correlated with the occurrence of complications such as intraoperative bleeding, postoperative leakage, anastomotic stenosis, and marginal ulcers [30]. Fourth, the large gastric capacity and wide anastomotic stoma of OAGB make it less likely to cause increased intragastric pressure [9].

Regarding late postoperative complications, our study showed no significant differences between the two procedures. In addition, when we added 24 cases of RYGB and 42 cases of OAGB that had surgical complications 2 years after surgery, which were separately mentioned in the study of Robert et al., the results of the meta-analysis still showed no significant differences between the two groups. Common complications of OAGB include nutritional deficiencies, gallstones, and diarrhea; common complications of RYGB include anastomotic ulcers, gallstones, and abdominal pain. In particular, Rober et al. reported nine malnutrition complications in OAGB. This is consistent with the conclusion that OAGB has more nutritional complications as mentioned in some studies, and the most common cause of nutritional deficiency in OAGB may be related to our presumed longer biliopancreatic limbs, while RYGB longer biliopancreatic limbs inhibited more nutrient absorption than OAGB [14, 40, 41]. The anastomotic ulcer of RYGB may also be related to the increase in the number of anastomotic stoma, and it is related to factors such as less vascular supply, difficult operation, and increased intragastric pressure due to small gastric volume.

### Comorbidity Remission

The remission of diabetes after the two surgical methods was *P* = 0.08, and there was no significant difference between the two; however, the remission of diabetes was still considered a high remission rate. In 2015, the International Diabetes Organization confirmed the good blood sugar control after metabolic surgery, and suggested that for diabetic patients with a BMI range of 30.0‒34.9 kg/m^2^, obesity surgery should be considered when blood sugar control is poor [42].

There were differences in postoperative hyperlipidemia remission in the included article analysis, and the remission rate after OAGB was higher. However, only two articles were included, and one of them accounted for 96.2% of the weight; therefore, the evidence was weak, and no differences in the management of hyperlipidemia between the two surgical methods were observed. Moreover, there was no significant difference in the postoperative remission rate of patients with hypertension, and both surgical methods were considered effective in improving obesity and hypertension.

Bariatric surgery was previously considered a viable option for the treatment of morbidly obese patients with refractory GERD [43]. In our study, we found no significant difference in GERD remission; however, the sample size was small, included literature were few, evidence strength was weak, and conclusion questionable. RYGB improves some patients’ symptoms over time; however, the sample size was small and there are many subjective indicators; hence, the conclusion is not adequately reliable [21]. The study by Eskandaros et al. showed that the alkaline reflux resulting from RYGB was significantly lower than that from OAGB only at 6 and 12 months, while the GERD, percentage of esophageal acid exposure time, and number of acid reflux were significantly improved; however, there were no significant differences between them [25]. Analysis of the reasons for the improvement of alkaline reflux in the initial stage of RYGB may be due to the establishment of a bypass and the direct entry of biliopancreatic juice into the jejunal output loop, which is not directly connected with the gastric cavity. Moreover, RYGB can be used as a surgical option for obese patients with GERD symptoms [44]. However, in the long run, the treatment of GERD using OAGB is closely related to the long gastric pouch, large gastric volume, and wide anastomotic stoma, which can reduce the intragastric pressure or increase the gastroesophageal pressure gradient [30, 31, 45]. Because the gastric pouch is long, the alkaline body fluid in the intestine is not easily refluxed to the cardia and above. In addition, the gastric volume is large, and when there is an increased pressure in the stomach, there is a lesser chance of the food in the stomach flowing back into the esophagus. The width of the anastomotic stoma will not cause reflux of gastric contents into the esophagus because of the restricted discharge of gastric contents. Therefore, in our analysis, OAGB is no worse than RYGB for GERD remission.

### Operation Time, Revision, and Quality of Life

Seven articles were included in a comparative analysis of the length of surgery and it was concluded that the operation time of OAGB was significantly shorter than that of RYGB. The heterogeneity of the analysis of the length of operation was relatively high. We performed the heterogeneity analysis by excluding articles apiece, and the study remained statistically significant after excluding the articles with high heterogeneity. The high heterogeneity screened out may be the reason why the mean operation time of this surgical method differs from the mean operation time of other articles.

Only one article that was included had postoperative revision, and the quality of the evidence was insufficient to effectively compare the two surgical modalities. In some obese patients with poor weight control, bile reflux, anastomotic ulcers, and other complications after other surgical methods, OAGB and RYGB can be used as revision methods [46]. Moreover, patients who need revision surgery after OAGB can continue with RYGB [40, 47]. However, patients who need a revision of RYGB surgery can only be changed to distal RYGB surgery or biliopancreatic diversion with duodenal switch (BPD-DS) [48].

In addition, Rober et al. used the Bariatric Analysis and Reporting Outcome System (BAROS) and the Impact of Weight on Quality of Life (IWQOL) to assess quality of life; Katayama et al. used the medical outcomes of a 36-item short-form health survey (SF-36) to assess quality of life; and Lee et al. used the Gastro-Intestinal Quality of Life Index (GIQLI) to assess the quality of life. All three articles indicated that the quality of life after the two surgeries was significantly improved compared to before the surgery; however, there was no significant difference between them. In addition, in the study of Ibrahim et al., GIQLI was used to assess the quality of life, and it was believed that the quality of life in the RYGB group was higher than that in the OAGB group at 6 and 12 months after surgery. However, since the methodology of quality of life evaluation in several articles is not uniform, and there is a large bias in reading one article alone, it cannot be determined which type of postoperative quality of life is better.

### Limitation

Our meta-analysis has some limitations. First, the number of articles evaluating the effect of weight loss after surgery is sufficient; however, the different expressed indicators lead to fewer articles that can be included in data analysis at various time points, which weakened the quality of the evidence. We hope that adding more articles in the future will improve the strength of the evidence, or suggest that researchers unify the indicators of postoperative weight loss. Second, some articles did not mention blinding, which may lead to an unreliable assessment of risk of bias. Third, the large difference in postoperative follow-up time resulted in fewer articles included in the analysis, which influenced the results of the analysis. Finally, the postoperative follow-up time was short; hence, no conclusion could be drawn on the treatment results of the sustained effect of weight loss and postoperative complications.

## Conclusion

OAGB is not inferior to RYGB in terms of weight loss and comorbidity remission, and the lower BMI in the 1st and 2nd years after OAGB may be related to the longer biliopancreatic limbs. The operative time of OAGB is significantly shorter than that of RYGB, and both surgery methods have a very low revision rate. There are fewer early complications after OAGB, and we found more anastomotic leaks with RYGB. There were no significant differences in intraoperative and late complications between the two surgery methods; however, we found that OAGB has a higher incidence of malnutrition. Our findings suggest that there is no obvious advantage or disadvantage in terms of weight loss effect between the two; however; more large-sample and long-term randomized controlled trials are needed to verify this. Therefore, further research is needed on the specification of biliopancreatic limb length and long-term complication rates.

## Data Availability

Data openly available in a public repository.
